# A case report of *Herbaspirillum* infection in rural Australia

**DOI:** 10.1128/asmcr.00091-24

**Published:** 2025-03-28

**Authors:** Elizabeth Josy Panikulam, Aideanna Seenarain, Himal Shrestha, Jordan Leung Wan Chin, Tasrifa Jahan, Grace Yap, Andrew Strack, Norelle L. Sherry, Zaal Meher-Homji

**Affiliations:** 1 Department of Infectious Diseases, Latrobe Regional Health, Victoria, Australia; 2 Department of Microbiology, Dorevitch Pathology, Victoria, Australia; 3 Department of Microbiology and Immunology, Microbiological Diagnostic Unit Public Health Laboratory, University of Melbourne, at the Peter Doherty Institute for Infection and Immunity, Melbourne, Victoria, Australia; 4 Department of Infectious Diseases and Immunology, Austin Health 3805, Heidelberg, Victoria, Australia; 5 School of Rural Health, Monash University, Victoria, Australia; Vanderbilt University Medical Center, Nashville, Tennessee, USA

**Keywords:** *Herbaspirillum*, MALDI-TOF MS, whole genome sequencing, Vitek 2, 16S PCR

## Abstract

**Background:**

*Herbaspirillum* is a genus of Gram-negative bacilli typically found in soil, which has recently been recognized as a cause of opportunistic infections in humans, affecting both immunocompromised and immunocompetent individuals.

**Case Summary:**

We report two patients from rural Australia who developed infections caused by two distinct *Herbaspirillum* species and a review of the literature of human infections caused by this organism. These isolates were misidentified on the Vitek 2 system as *Burkholderia cepacia* and were identified to the genus level with matrix-assisted laser desorption/ionization time-of-flight mass spectrometry (MALDI-TOF) and 16S PCR. Accurate species identification was confirmed with whole genome sequencing analysis.

**Conclusion:**

*Herbaspirillum* species are increasingly identified as human pathogens, which can be accurately identified to the species level with sequencing techniques.

## INTRODUCTION

*Herbaspirillum* species are Gram-negative, oxidase-positive nonfermenting bacilli that are ubiquitously present in the natural environment including water, soil, and plants (1). Human infections have been increasingly reported with modern molecular diagnostic methods, including matrix-assisted laser desorption/ionization time-of-flight mass spectrometry (MALDI-TOF MS) and novel sequencing techniques (2).

We report two cases of *Herbaspirillum* bacteremia in rural Australia that presented to our institution, a regional referral center in eastern Victoria. We discuss microbiological methods of identifying the organism and subsequently summarize the prior literature on human infection due to this organism.

## CASE PRESENTATION

### Case 1

A 75-year-old male presented 3 months post-wedge resection of stage T3N0M0 left lower lobe lung adenocarcinoma and was admitted with fever, dyspnea, and a non-productive cough 1 day following his third cycle of adjuvant chemotherapy (carbotaxel and pemetrexed, commenced 2 months prior). He reported being an avid gardener, particularly of his fruit trees.

**Fig 1:**
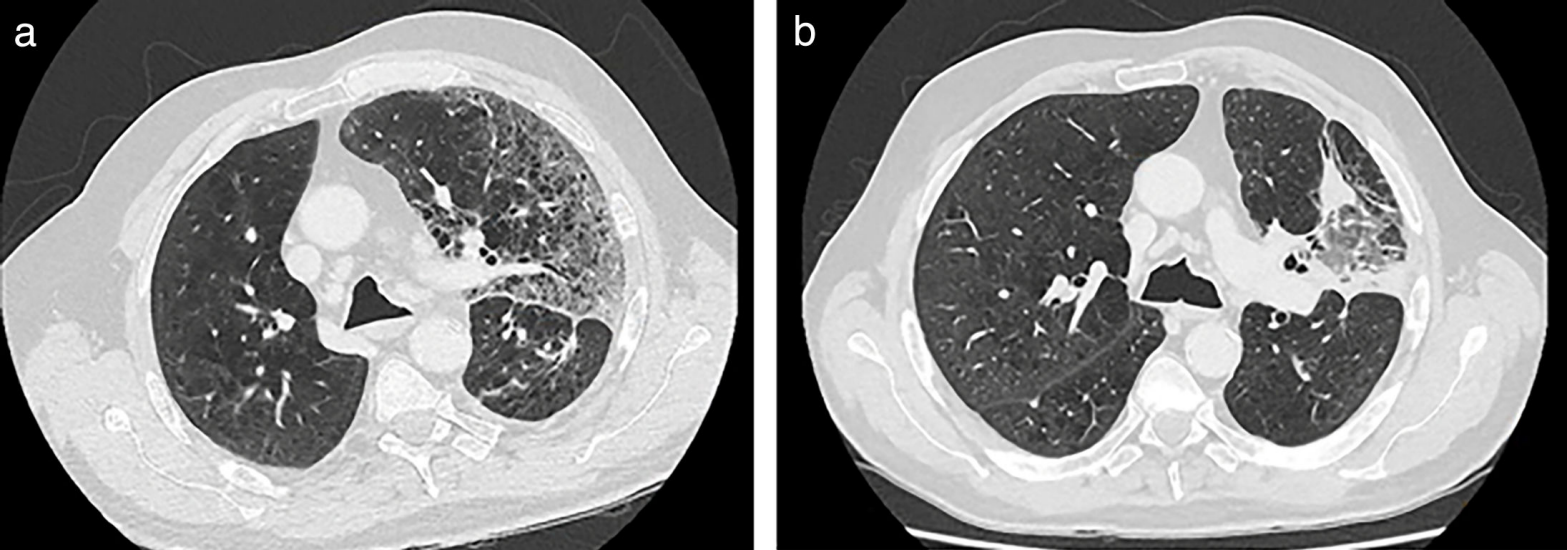
(a) Patient 1, CT chest pre-treatment. Diffuse ground-glass changes with interlobular septal thickening in left upper lobe, indicative of acute inflammation or infection. (b) Patient 1, CT chest post-treatment. Improvement in ground-glass opacities, traction bronchiectasis, and scarring in the posteroinferior aspect of the left upper lobe.

On arrival, he showed features of severe sepsis (temperature, 39.2°C; blood pressure, 86/60 mmHg; heart rate, 106 bpm, oxygen saturation [SpO2], 88% on room air; Glasgow coma scale of 14). Laboratory investigations demonstrated neutrophils of 1.2 × 10^9^/L, a C-reactive protein (CRP) of 220 mg/L, and a normal serum lactate of 1.3 mmol/L. Computed tomography (CT) of the chest demonstrated new diffuse ground-glass changes in the left upper lobe with interlobular septal thickening, consistent with an acute inflammatory or infective process (Fig. 1a), with expected left lower zone post-surgical changes and no local recurrence of malignancy. Blood cultures were drawn, and he commenced on empirical intravenous piperacillin/tazobactam 4.5 g every 6 h for neutropenic sepsis secondary to severe pneumonia.

**Fig 2:**
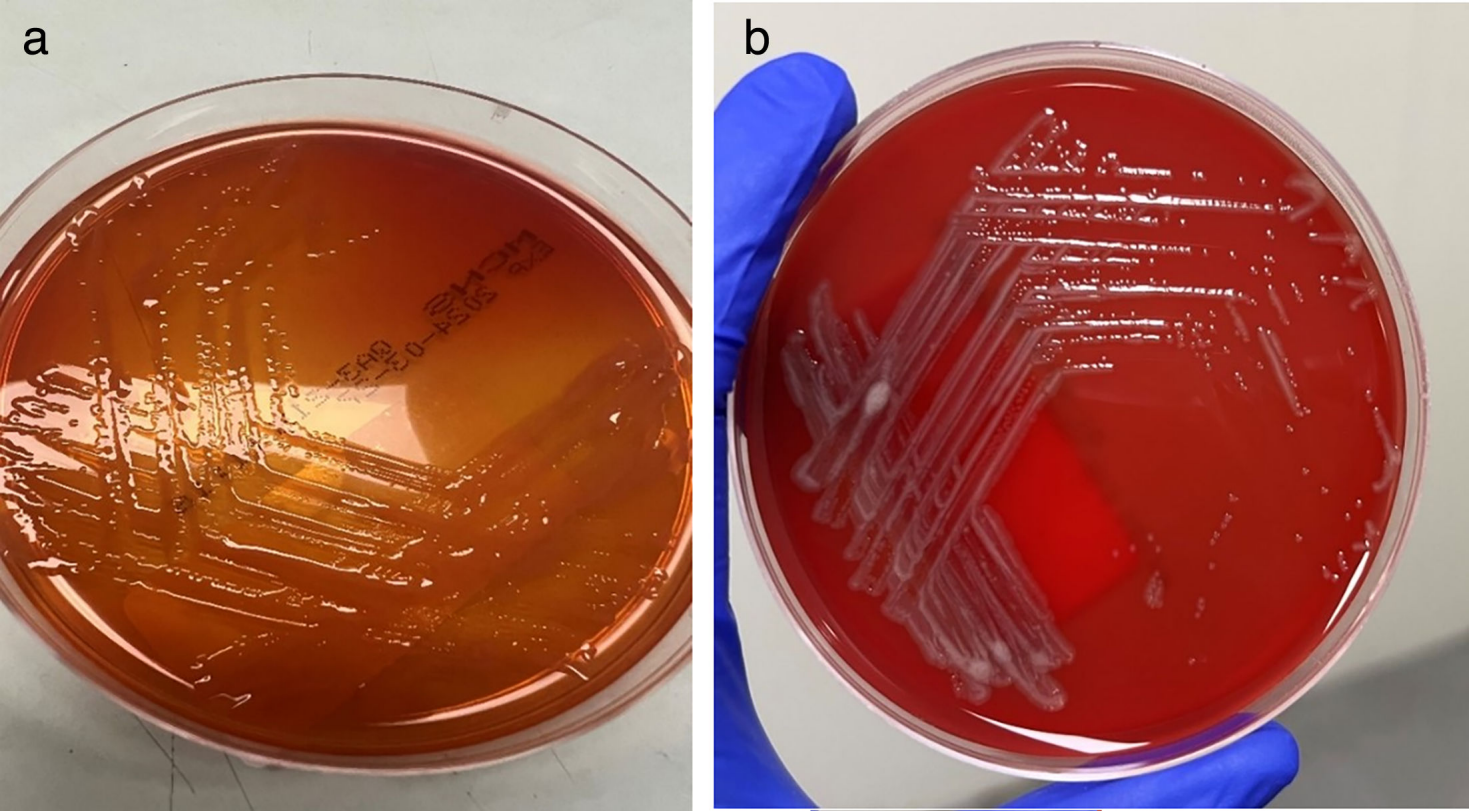
(a) Growth on MacConkey agar at 24 h. (b) Growth on (horse blood agar (HBA) at 24 h.

Blood culture samples were taken and loaded onto the bioMerieux BACTI/ALERT VIRTUO blood culture system. The aerobic bottle flagged positive within 24 h of incubation. The Gram stain microscopy showed Gram-negative bacilli. Samples were plated onto horse blood agar (HBA) with a vancomycin disc, chocolate agar (CHOC), MacConkey agar (MAC), and incubated at 37°C in 5% CO_2_. An anaerobic blood agar (ANA) plate was inoculated, stamped with a metronidazole disc, and incubated anaerobically using a Thermo Fisher Oxoid Anaerobic culture pack at 37°C. All culture plates were read after a 10 h incubation and showed growth of small gray non-hemolytic colonies on HBA (Fig. 2b). The organism was vancomycin resistant, non-lactose fermenting (Fig. 2a), and a strict aerobe. On initial bench testing, the organism was oxidase positive and indole negative. MALDI-TOF MS (bioMerieux VITEK MS PRIME Clinical use v 3.2 database, bioMérieux) identified the organism as *Herbaspirillum huttiense*. The VITEK 2 GN card (identification knowledge base, Systems version 09.00.014, bioMerieux) gave a 95% probability of *Burkholderia cepacia* group. To confirm the identification, the isolate was referred for 16S ribosomal RNA (rRNA) sequencing, which identified the organism as *H. huttiense*. This patient had four further positive sets of blood cultures over a 7-day period with the same organism.

The patient remained persistently febrile while continuing piperacillin/tazobactam. On day 6 of admission after preliminary organism identification, intravenous meropenem 1 g every 8 h was commenced for severe pneumonia in an immunocompromised host. Within 24 h of commencing meropenem, he began to defervesce with reduction in the CRP. He was discharged home 4 days later on high-dose oral ciprofloxacin 750 mg every 12 h for a total antibiotic course of 4 weeks. Three months after the completion of therapy, the patient remains well with no recurrence of his symptoms and CT resolution of left upper lung infective changes (Fig. 1b).

### Case 2

A 57-year-old female with a history of intravenous substance abuse presented with complaints of diaphoresis and intermittent chest pain associated with productive cough and dyspnea. She had a significant past medical history of an atrial septal defect (ASD) occluder device inserted 17 years ago.

Physical examination revealed a systolic murmur radiating to the carotids. The patient’s vital signs were within normal limits. Laboratory investigations showed elevated neutrophils at 12.5 × 10⁹/L, a CRP of 126 mg/L, and a normal serum lactate of 1.8 mmol/L. Blood cultures were drawn, and empirical intravenous gentamicin and vancomycin were initiated for suspected infective endocarditis.

**Fig 3:**
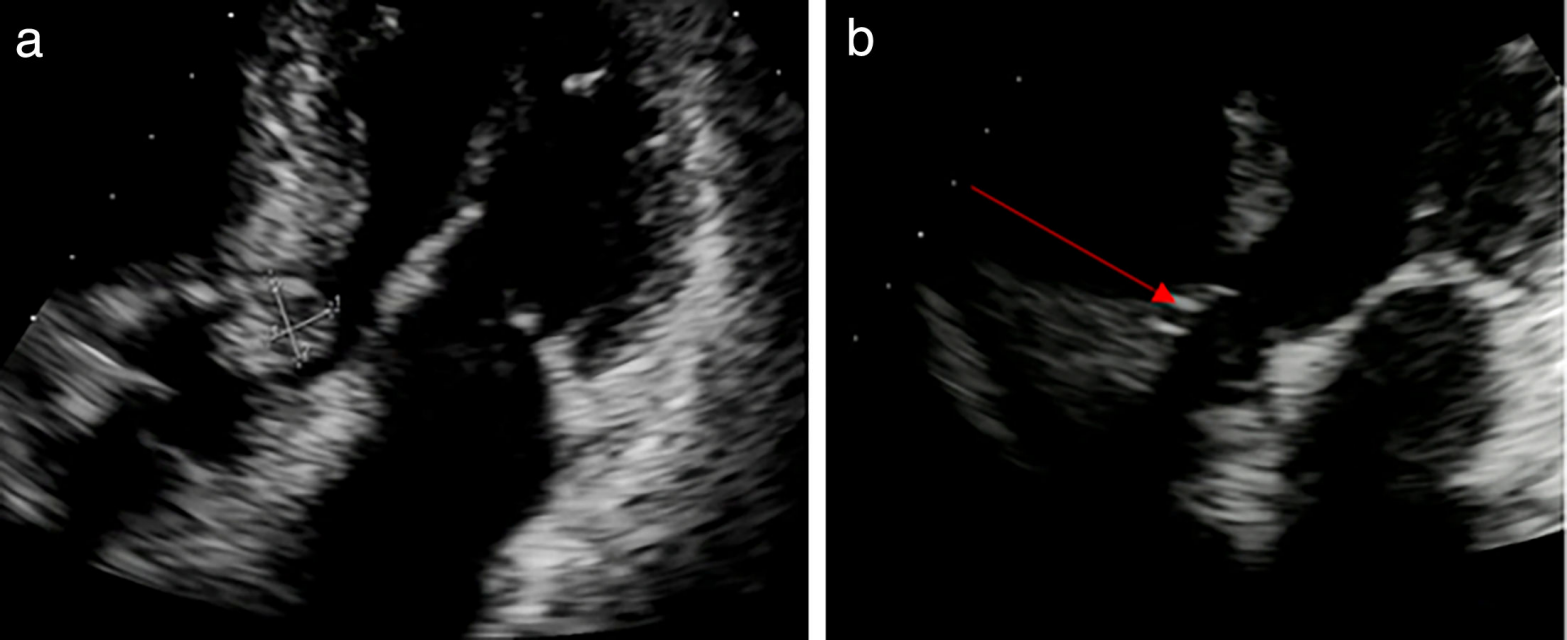
(a) Patient 2, transthoracic echocardiogram pre-treatment. A 1 × 1.2 cm echogenic mass on the aortic valve with associated moderate aortic stenosis and aortic regurgitation in keeping with a vegetation. (b) Patient 2, transthoracic echocardiogram post-treatment. Well-seated aortic valve bio-prosthesis with no regurgitation (arrow).

A CT pulmonary angiogram showed changes consistent with chronic obstructive pulmonary disease (COPD) with signs suggestive of pulmonary hypertension and patchy ground-glass opacities indicative of inflammation or pulmonary edema. Transthoracic echocardiogram (TTE) revealed a mildly dilated left atrium, an ASD closure device with no residual shunting, and a 1 × 1.2 cm mobile echogenic mass on the left coronary cusp of the aortic valve (Fig. 3a). There was moderate aortic stenosis and regurgitation, with the left ventricular ejection fraction (EF) visually estimated at >75%.

This patient had four blood culture sets taken over a 2-day period. All bottles were loaded on to the bioMerieux BACTI/ALERT VIRTUO system. The aerobic bottle of all sets flagged positive within 24 h incubation. Gram stain microscopy showed Gram-negative bacilli, and the cultures were set up as previously described in case one. The culture plates were read at 16–18 h incubation and showed a vancomycin-resistant, non-lactose fermenting strict aerobic organism (Fig. 2a). Bench testing showed it to be oxidase positive and indole negative. MALDI-TOF MS identified the organism as *H. huttiense*, and the VITEK2 GN card gave a 99% probability of *Burkholderia cepacia* group. The organism was polymyxin B resistant. To confirm the identification, the isolate was referred for 16S rRNA sequencing, which identified the organism as *H. huttiense*.

The patient underwent tissue aortic valve replacement and opportunistic coronary artery bypass grafting (CABG). Perioperatively, she was commenced on intravenous meropenem 1 g every 8 h. She received meropenem for 2 weeks, followed by intravenous ertapenem 1 g daily, for a total of 6 weeks of intravenous antibiotics. Six weeks after completing intravenous antibiotic therapy, the patient remained well without symptom recurrence. A follow-up TTE showed a well-seated aortic bioprosthesis with satisfactory Doppler parameters and no regurgitation (Fig. 3b).

### Antimicrobial susceptibility testing

Isolates underwent antimicrobial susceptibility testing using the Thermo Fisher Scientific Sensititre system. Minimum inhibitory concentrations (MICs) were interpreted using CLSI M100 Performance Standards for Antimicrobial Susceptibility Testing 34th Edition, Table 2B-5 MIC Breakpoints for Other Non-Enterobacterales (3).

Both isolates were susceptible to the third-generation cephalosporins (ceftriaxone and ceftazidime), the carbapenems (imipenem, meropenem), as well as aztreonam, levofloxacin, minocycline-aztreonam, levofloxacin, minocycline, piperacillin-tazobactam, and trimethoprim-sulfamethoxazole (Table 1). Isolate 1 was susceptible to ciprofloxacin, while isolate 2 was intermediate. For the aminoglycosides, both isolates displayed susceptibility to amikacin and tobramycin, while isolate 1 was intermediate to gentamicin, and isolate 2 was susceptible. While no interpretive guidelines exist for colistin, both isolates displayed high MICs (>16 mg/L).

**TABLE 1:** Antimicrobial susceptibility results^*a*^

Antimicrobial	Isolate 1		Isolate 2	
	MIC (mg/L)	MIC interpretation	MIC (mg/L)	MIC interpretation
Aztreonam	4	S	8	S
Cefepime	≤1	S	≤1	S
Ceftazidime	≤1	S	≤1	S
Ceftriaxone	≤0.5	S	1	S
Ciprofloxacin	1	S	2	I
Levofloxacin	≤1	S	2	S
Imipenem	≤1	S	≤1	S
Meropenem	≤0.12	S	≤0.12	S
Piperacillin-tazobactam	≤4	S	≤4	S
Tetracycline	≤4	S	≤4	S
Minocycline	≤1	S	≤1	S
Trimethoprim-sulfamethoxazole	≤2	S	≤2	S
Amikacin	16	S	4	S
Gentamicin	8	I	≤2	S
Tobramycin	2	S	1	S
Colistin	>16	NG	>16	NG
				

^*a*^ S, susceptible; I, intermediate; R, resistant; NG, no interpretive guidelines.

### Whole genome sequencing (WGS) analysis

Genomic DNA was extracted from bacterial isolates using a JANUS automated workstation (PerkinElmer), employing Chemagic magnetic bead technology (PerkinElmer). Library preparation was performed using the Nextera XT DNA Library Preparation Kit (following the manufacturer’s instructions, Illumina Inc.). Sequencing was performed on an Illumina next-generation sequencing platform (NextSeq 500/550), generating short reads with 2 × 150 bp paired-end chemistry as described here (4). In addition to the whole genome sequencing (WGS) analysis, the sequence data were also used for 16S rRNA sequencing. The 16S rRNA gene sequences were bioinformatically extracted from the WGS data using barrnap (version 0.9, https://github.com/tseemann/barrnap), a tool designed for rapid prediction of rRNA genes from genomic sequences. The extracted 16S rRNA sequences were queried using NCBI’s BLASTN search tool (https://blast.ncbi.nlm.nih.gov/Blast.cgi) against the standard nucleotide (nr/nt) database (5). The BLASTN results were filtered to exclude uncultured bacteria for taxonomic identification.

WGS analysis provided high-resolution species identification for the isolates from both patients. This was initially conducted to determine if the isolated pathogens among the two cases were genomically related. Using k-mer-based taxonomic classification (Kraken2 v2.1.2 [6, 7] with the GTDB database, the isolate from isolate 1 was identified as *Herbaspirillum seropedicae* (73.89% sequence reads classified), while the isolate from isolate 2 was classified as *Herbaspirillum aquaticum* (81.88% sequence reads classified). To confirm these classifications, we performed phylogenetic analysis using genetic distances (mashtree v1.2.0[8, 9]) against representative genomes of the *Herbaspirillum* genus from the RefSeq database, including *H. seropedicae* (*n* = 7), *H. aquaticum* (*n* = 2), and *H. huttiense* (*n* = 7). The lowest genetic distance and closest phylogenetic placement support the initial classifications as *H. seropedicae* and *H. aquaticum* and for isolates 1 and 2, respectively.

## DISCUSSION

*Herbaspirillum* species are a rare cause of human infection, which is increasingly being reported with modern microbiological identification techniques. It belongs to the class of Betaproteobacteria of the order Burkholderiales, which includes *Burkholderia* species, *Ralstonia* species, and other endophytic bacteria (10). Like other environmental Gram-negative organisms, it can cause a variety of clinical syndromes and can be resistant to the empirical antimicrobials used to treat these syndromes. Laboratory misidentification of *Herbaspirillum* species can also occur, particularly with Vitek 2 systems (2). Here, we report two cases of bacteremia with different clinical characteristics and summarize the available literature.

Table 2 summarizes the 16 published cases of *Herbaspirillum* species infections in humans. Human infection has occurred across a wide age and geographic range. The vast majority of patients have predisposing medical conditions, such as immunocompromised states, respiratory or cardiac disease, and diabetes mellitus. In keeping with the known niche of the organism, several reported cases had recent close contact with soil, farms, or contaminated environments prior to hospital admission (11–13). Additionally, some cases involve nosocomial infections and outbreaks mainly due to catheter-related bloodstream infections (14, 15). The clinical spectrum of infection ranges from bloodstream infections (57%), pneumonia (50%), catheter-related bloodstream infections (21.4%), and less commonly, infective endocarditis (7.1%) (16, 17).

**TABLE 2:** Summary of reported cases of *Herbaspirillum* species infections in humans

Case	Reference	Age/sex	Country	Predisposing conditions	Clinical syndrome	Identification method	Treatment	Outcome
1	Spilker et al. (12)	26/M	USA	Cystic fibrosis with severe lung disease, pancreatic insufficiency, and liver disease	Pneumonia/bacteremia	Blood C/S on selective medium and 16S rRNA PCR	IV ceftazidime + tobramycin + oral trimethoprim/sulfamethoxazole (TMP-SMX) + levofloxacin + minocycline × 4 weeks	Survived
2	Chen et al. (13)	48/F	China	Acute lymphoblastic leukemia	Bacteremia	16S rRNA PCR and gene sequencing	Cefmetazole + gatifloxacin	Survived
3	Regunath et al. (14)	46/M	USA	Asthma	Pneumonia	MALDI-TOF MS and 16S rRNA gene sequencing	Piperacillin/tazobactam + doxycycline × 14 days	Survived
4	Berardino et al. (15)	59/F	Spain	Essential thrombocythemia, diabetes mellitus (DM)	Pneumonia	MALDI-TOF MS and 16S rRNA gene sequencing	Piperacillin/tazobactam	Survived
5	Hernandez et al. (16)	2m	Spain	Premature birth at 26 weeks, patent ductus arteriosus	Pneumonia/bacteremia/central line-associated bloodstream infection (CLABSI)	MALDI-TOF MS	Meropenem	Survived
6	Tas M et al. (18)	1m	USA	IV methylprednisolone for thrombocytopenia	Bacteremia	MALDI-TOF MS	Piperacillin/tazobactam + tazobactam + vancomycin for 10 days	Survived
7	Liu et al. (19)	93/M	South Korea	Advanced age	Pneumonia	MALDI-TOF MS and 16S rRNA gene sequencing	Meropenem + colistin switched toceftazidime + minocycline then TMP-SMX	Died
8	Uzuner et al. (20)	54/M	Turkey	Acute myeloid leukemia on chemotherapy	Bacteremia	MALDI TOF MS	Meropenem	Survived
9	Bloise et al. (17)	64/M	Spain	Brain space-occupying lesion, large cell lung cancer	Pneumonia	MALDI TOF MS	Meropenem × 10 days	Died
10	Bloise et al. (17)	64/F	Spain	Breast cancer on chemotherapy	Bacteremia/CLABSI	16S rRNA PCR and gene sequencing	Piperacillin/tazobactam × 15 days	Survived
11	Bloise et al. (17)	38/M	Spain	Polytrauma	Bacteremia/ CLABSI	16S rRNA PCR and gene sequencing	Meropenem × 2 days deescalated to piperacillin/tazobactam × 11 days	Survived
12	Gungor et al. (21)	11/F	Turkey	Tibial osteosarcoma on chemotherapy	Right-sided infective endocarditis (IE)	Vitek MS	Meropenem + amikacin × 4 weeks	Survived
13	Li et al. (2)	72/M	China	N/A^*a*^	Bacteremia	MALDI TOF MS and 16S rRNA gene sequencing	Meropenem + tigecycline switched to moxifloxacin and piperacillin/tazobactam	Survived
14	Ruiz de Villa et al. (10)	59/F	USA	DM, chronic obstructive pulmonary disease (COPD)	Pneumonia/septic shock	MALDI TOF MS and 16S rRNA gene sequencing	Cefepime × 7 days then oral levofloxacin × 5 days	Survived
15	Case 1 (*H.seropedicae)*	75/M	Australia	Lung adenocarcinoma on chemotherapy	Pneumonia/bacteremia	MALDI-TOF MS and 16S rRNA gene sequencing	Meropenem × 4 days, then oral ciprofloxacin × 3 weeks	Survived
16	Case 2 (*H. aquaticum*)	57/F	Australia	IV drug user, ASD occlusion device, COPD, pulmonary arterial hypertension	Aortic valve IE	MALDI TOF MS and 16S rRNA gene sequencing	Meropenem × 2 weeks, switched to ertapenem × 4 weeks, then high-dose oral ciprofloxacin × 2 weeks	Survived

^*a*^ N/A, not applicable.

In most of the publications, patients have received multiple antibiotics, the most common being meropenem (50%) and piperacillin/tazobactam (42.8%) (2, 11, 14). In case 1, despite the cultures indicating sensitivity to piperacillin-tazobactam and meropenem, the patient’s fever subsided only after the initiation of meropenem. In most of the reported cases, patients initially received piperacillin/tazobactam for broad Gram-negative coverage, with subsequent escalation to meropenem. While some patients responded well to piperacillin/tazobactam, in our case, clinical improvement required carbapenems.

Both isolates tested susceptible to a range of antimicrobials including third-generation cephalosporins (ceftriaxone and ceftazidime), the carbapenems (imipenem, meropenem) as well as aztreonam, levofloxacin, minocycline, and trimethoprim-sulfamethoxazole. These antimicrobial susceptibility findings were consistent with other published data (17) and suggest that *Herbaspirillum* species are generally not multidrug resistant. High colistin MICs have also been previously reported, suggesting this organism may be intrinsically resistant.

In previous publications, it has been noted that some patients received a short course of 10–14 days of antibiotics, while others had a protracted course for up to 4 weeks (2, 20). We transitioned our patients to high-dose oral ciprofloxacin (750 mg twice daily) for 3 weeks in case 1 and 2 weeks in case 2 upon discharge with good effect. The duration of antibiotics was guided by the source of infection. It does not appear that infections secondary to *Herbaspirillum* species tend to relapse after appropriate susceptible antimicrobials. Out of the cases documented in publications, there were only two fatalities (16, 21). One was a 93-year-old male who was frail and subsequently palliated, while the other patient had concurrent methicillin-resistant *Staphylococcus aureus* (MRSA) bacteremia, potentially impacting their prognosis. The remaining patients responded well to antibiotic therapy and recovered successfully from the infection.

In both our cases, the Vitek 2 Gram-negative identification system misidentified the organisms as *Burkholderia cepacia*. Although the Vitek 2 system gave an excellent identification score in both cases, it also flagged that the urea was positive for both isolates. This was confirmed by inoculation of the isolates onto urea slopes and incubated for 24–72 h. As our laboratory routinely uses MALDI-TOF MS, we were able in both cases to identify *H. huttiense* (which was accurate to at least the genus level), and this finding was replicated by 16S rRNA sequencing. It is important to note that the Vitek 2 system and the MALDI-TOF MS system have a different range of organisms on their respective databases. This has given rise to the discrepancy seen in organism identification using both methods of identification. Given that *B. cepacia* isolates are urease negative, in the absence of MALDI-TOF MS, it is possible that this simple test could be used as an indicator that the organism has been misidentified by Vitek 2 as *B. cepacia*, and further examination of the isolate is necessary. Another suggestion has been to use susceptibility patterns to differentiate *B. cepacia* from *Herbaspirillum* species, as the former usually exhibits multidrug resistance (2).

WGS analysis demonstrated superior resolution in species identification compared with MALDI-TOF and 16S rRNA sequencing methods. Importantly, the genomic evidence revealed that the isolates belong to different species, indicating that they were not genomically related and were unlikely to originate from the same source. This finding highlights the role of WGS in providing accurate and detailed microbial identification, which is essential for determining infection etiology, establishing or ruling out transmission links, and guiding evidence-based clinical management strategies (7, 9, 22).

*Herbaspirillum* species share many characteristics with other genera of plant-associated bacteria, such as *Ralstonia*, *Ochrobactrum*, *Achromobacter*, *Burkholderia*, and *Pseudomonas* species (1). Due to the difficulty of clinical laboratories in identifying these bacteria based on phenotypic methods, with the advent of MALDI-TOF MS and WGS becoming widely used in routine diagnostic laboratories, we may see an increase in the accurate identification of *Herbaspirillum* species from clinical specimens. Improved identification methods and increased awareness of this bacterium will help us to better understand its prevalence in the community and its role in human infections.

### Conclusion

*Herbaspirillum* species are rare Gram-negative environmental pathogens that are increasingly recognized as a cause of human infections. The most common clinical manifestation is bloodstream infection and pneumonia. The organism can be misidentified on the Vitek 2 system as *Burkholderia cepacia* and is accurately identified to the genus level by MALDI-TOF or 16S PCR. WGS demonstrates superior resolution to a species level.

## Data Availability

Sequence data generated for this study are publicly available in the GenBank Sequence Read Archive (SRA) under BioProject PRJNA870170.
